# Hypovitaminosis A: a hidden cause of neurotrophic keratitis

**DOI:** 10.5935/0004-2749.2023-0323

**Published:** 2024-04-03

**Authors:** Eduardo Melani Rocha, Diego Rocha Gutierrez

**Affiliations:** 1 Department of Ophthalmology, Faculdade de Medicina de Ribeirão Preto, Universidade de São Paulo, Ribeirão Preto, SP, Brazil

Re: Wang EY, et al.: Global Trends in Blindness and Vision Impairment Resulting from Corneal Opacity 1984-2020: a meta-analysis (Ophthalmology. 2023; 130(8): 863-871)

To the Editor:

Wang et al.^(1)^ presented a comprehensive meta-analysis of blindness and vision impairment resulting from corneal opacity. Wang et al. stated that the “major causes of corneal opacification include trachoma, infectious keratitis, xerophthalmia, use of traditional eye medicines, and ocular trauma”. Grouping these conditions revealed the amount and complexity of corneal blindness worldwide, which was warranted and identified at a crucial time. The discussion highlighted the likely role of vitamin A deficiency in corneal opacity.

Vitamin A deficiency is detrimental to corneal transparency. However, the understanding of the mechanisms of hypovitaminosis A in keratomalacia remains poor. Furthermore, it is inaccurate to assume that reduced tear flow is the sole cause of keratomalacia. A study published in 1934 by Mellanby demonstrated a probable neurotrophic origin for vitamin A deficiency-associated corneal opacity^(2)^. In the study, vitamin A deprivation in rats not only induced keratomalacia but also revealed trigeminal ganglion degeneration in the histological sections. These findings support the hypothesis that vitamin A deficiency induces keratomalacia via sensorial denervation of the cornea. Therefore, hypovitaminosis A is a subtype of neurotrophic keratitis. Xerophthalmia is estimated to blind half a million children each year^(3)^. Considering this mechanism, hypovitaminosis A and its clinical spectrum may count as risk factors and triggers for xerophthalmia and corneal opacities.

We would like to congratulate Wang et al. for this narrative review of corneal opacities. Additionally, we would like to propose that based on previous observations of xerophthalmia causing keratomalacia and corneal blindness due to hypovitaminosis A of different origins that xerophthalmia may be a subtype of neurotrophic keratitis. This may be attributed to the fact that vitamin A deprivation causes trigeminal ganglion degeneration.
